# A Post-Haustorial Defense Mechanism is Mediated by the Powdery Mildew Resistance Gene, *PmG3M*, Derived from Wild Emmer Wheat

**DOI:** 10.3390/pathogens9060418

**Published:** 2020-05-28

**Authors:** Zhen-Zhen Wei, Valentyna Klymiuk, Valeria Bocharova, Curtis Pozniak, Tzion Fahima

**Affiliations:** 1 Institute of Evolution, University of Haifa, 199 Abba-Hushi Avenue, Mt. Carmel, 3498838 Haifa, Israel; zhenzhen-wei@hotmail.com (Z.-Z.W.); valentyna.klymiuk@usask.ca (V.K.); bocharovavr@gmail.com (V.B.); 2 The Department of Evolutionary and Environmental Biology, University of Haifa, 199 Abba-Hushi Avenue, Mt. Carmel, 3498838 Haifa, Israel; 3 Crop Development Centre and Department of Plant Sciences, University of Saskatchewan, 51 Campus Drive, Saskatoon, SK S7N 5A8, Canada; curtis.pozniak@usask.ca

**Keywords:** powdery mildew, wild emmer wheat, *PmG3M*, post-haustorial resistance, H_2_O_2_ accumulation, programmed cell death

## Abstract

The destructive wheat powdery mildew disease is caused by the fungal pathogen *Blumeria graminis* f. sp. *tritici* (*Bgt*). *PmG3M*, derived from wild emmer wheat *Triticum dicoccoides* accession G305-3M, is a major gene providing a wide-spectrum resistance against *Bgt*. *PmG3M* was previously mapped to wheat chromosome 6B using an F_6_ recombinant inbred line (RIL) mapping population generated by crossing G305-3M with the susceptible *T. durum* wheat cultivar Langdon (LDN). In the current study, we aimed to explore the defense mechanisms conferred by *PmG3M* against *Bgt.* Histopathology of fungal development was characterized in artificially inoculated leaves of G305-3M, LDN, and homozygous RILs using fluorescence and light microscopy. G305-3M exhibited H_2_O_2_ accumulation typical of a hypersensitive response, which resulted in programmed cell death (PCD) in *Bgt*-penetrated epidermal cells, while LDN showed well-developed colonies without PCD. In addition, we observed a post-haustorial resistance mechanism that arrested the development of fungal feeding structures and pathogen growth in both G305-3M and resistant RIL, while LDN and a susceptible RIL displayed fully developed digitated haustoria and massive accumulation of fungal biomass. In contrast, both G305-3M and LDN exhibited callose deposition in attempt to prevent fungal invasion, supporting this as a mechanism of a basal defense response not associated with *PmG3M* resistance mechanism per se. The presented results shed light on the resistance mechanisms conferred by *PmG3M* against wheat powdery mildew.

## 1. Introduction

Powdery mildew diseases of plants are widespread in temperate climates and are a continuous threat to agriculture as they evolve quickly to overcome plant resistance [1]. More than 650 species of powdery mildew fungi that can infect over 10,000 plant species were identified [2]. These plant pathogens are obligate biotrophs that can infect leaves, stems, flowers and fruits and frequently decrease the grain yield and quality of agricultural crops [1,3,4]. *Blumeria graminis* is a filamentous ascomycete parasite fungus infecting *Poaceae* species including cereal crops, such as wheat (*Triticum aestivum*) and barley (*Hordeum vulgare*) [4]. *B. graminis* f. sp. *tritici* (*Bgt*) is specific to wheat, while *B. graminis* f. sp. *hordei* (*Bgh*) is specific to barley, but their disease cycles are highly similar [5,6]. The infection process of the disease, during the asexual stage, includes the following steps and processes: (i) inoculation a haploid conidium of *Bgt* is landing on leaf of a susceptible wheat plant; (ii) germination – the spore is germinating and forming first primary germ tube(s) that would never fully penetrate into epidermal cells, and then a secondary germ tube that will differentiate into an appressorial germ tube (AGT); (iii) penetration–AGT differentiates into a mature appressorium with a narrow protrusion called penetration peg, which directly penetrate the cell walls of host epidermis by means of turgor pressure and enzymatic activity; (iv) colonization the penetration peg is enlarged into a feeding structure called haustorium, formed within the host cell to absorb host nutrients and deliver effector proteins; (v) invasion – secondary hypha outgrowths are produced and generate penetration pegs that infect additional host cells; (vi) dissemination – following the invasion of powdery mildew, circular colonies with elongated and repeatedly branched hyphae are formed and spread epiphytically on the leaf surface. These colonies produce conidiophores that carry chains of conidia that are released to the environment to start new cycles of infection on the same or other plants. Young hyphae are transparent or whitish, while mature colonies are gray-brown color, sometimes showing dark dots that represent the cleistothecia fruiting bodies [5,6].

Plants protect themselves against pathogens by using a variety of chemical and physical defenses. For example, cell wall thickenings called papillae that are formed by deposition of callose, a (1,3)-β-glucan polymer, accumulated at the site of pathogen penetration, are effective physical barriers to slow pathogen invasion during plant–pathogen interactions [7]. In the early stage of defense responses induced by biotrophs, massive reactive oxygen species (ROS) accumulation can trigger a localized hypersensitive response (HR), a form of programmed cell death, which results in the limitation or even blocking of penetration and development of pathogen colonies, thus resulting in resistance to powdery mildew (reviewed by Hückelhoven [8]). The accumulation of H_2_O_2_ in the epidermal cells was demonstrated to play an important role in defense response of resistant host plants to fungal penetration in different plant–pathogen pathosystems, including wheat-*Bgt* [9].

*PmG3M* is a major resistance gene, derived from wild emmer wheat (WEW) *T. dicoccoides* accession G305-3M, that was shown to confer resistance to 54 *Bgt* isolates collected from different wheat species (e.g., *T. dicoccoides*, *T. durum* and *T. estivum*) in various countries representing four continents around the globe [10,11,12]. *PmG3M* was genetically mapped to the distal part of wheat chromosome arm 6BL using a mapping population generated from the cross of WEW G305-3M, the donor line of *PmG3M,* with the *Bgt* susceptible *T. durum* cv. LDN [12]. *PmG3M* was assigned to a chromosome region where no other *Pm* genes were localized in wheat before [12,13], suggesting it as a novel resistance gene, not found previously in the domesticated wheat germplasm. However, the resistance mechanism conferred by *PmG3M* is still unknown.

In the current study, we aimed to explore the defense mechanisms involved in the *PmG3M*-mediated resistance of wheat against *Bgt*. We focused on the characterization of fungal growth and development on susceptible versus resistant host in order to dissect the defense mechanism conferred by *PmG3M* from mechanisms of basal defense responses. We used advanced microscopy imaging technologies to characterize the compatible (susceptible) versus incompatible (resistant) host-parasite interactions at the cellular level of the wheat-*Bgt* pathosystem. We found that a post-haustorial resistance mechanism was activated by *PmG3M*, leading to arrest of the development of fungal feeding structures and termination of growth of the fungal biomass on the leaf surface. Programed cell death was also activated during the defense response of the host plant conferring the *PmG3M* gene, as evident by the accumulation of H_2_O_2_ in the *Bgt* penetrated epidermal cells of the resistant plants. On the other hand, callose deposition most likely represents a basal defense response, since it was detected in both, resistant and susceptible, wheat lines.

## 2. Results

### 2.1. Selection of Plant Material for Histological Studies and Verification of the Inheritance Pattern of the PmG3M Locus

**Figure 1:**
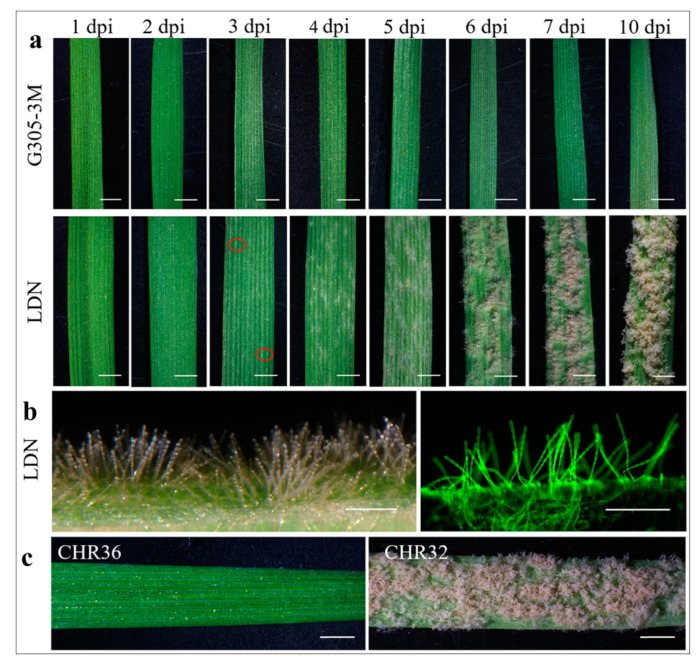
Macroscopic and microscopic observations of the resistant and susceptible host plants at different time points after *Bgt#70* inoculation. (**a**) Macroscopic observations of G305-3M and LDN at eight time points after *Bgt#70* inoculation. The first colonies of *Bgt* are marked with a red circle at 3 dpi on LDN; (**b**) colony observation on LDN at 5 dpi after *Bgt#70* infection. The *Bgt* colony on the left side was observed under light microscopy, while the colony on the right was observed under fluorescence microscopy after staining with wheat germ agglutinin (WGA); (**c**) macroscopic observations of the resistant RIL CHR36 and susceptible RIL CHR32 at 10 dpi. Scale bars: (**a,c**) 2 mm; (**b**) 250 μm.

In the current study, we have characterized the histological defense responses against *Bgt* in the parental lines G305-3M (resistant, IT = 0) and LDN (susceptible, IT = 4) (Figure 1a) and their F_6_ RIL progenies (CHR32 and CHR36), previously used for genetic mapping of *PmG3M.* The selected RILs carry reciprocal-like recombination events between markers *stm583* and *edm49* that flank the *PmG3M* locus (Supplementary Tables S1 and S2) and therefore they represent alternative parental alleles along this 6BL chromosome interval.

Furthermore, *Bgt* test showed that CHR36 is highly resistant (IT = 0, Figure 1c) to powdery mildew, while CHR32 is susceptible (IT = 4; Figure 1c), indicating that the reciprocal-like recombination events occurred between *PmG3M* and *stm583*. To verify the inheritance pattern of the *PmG3M* locus, we have tested 265 F_2_ plants, from the cross between G305-3M and LDN, for resistance to isolate *Bgt*#70. The segregation ratio of the phenotypic response to *Bgt* in this F_2_ population was 3.34R:1S (χ2 = 0.5547, *p* = 0.4564; Supplementary Table S3), therefore providing further evidence that the *PmG3M* resistance is conferred by a single dominant gene, derived from WEW accession G305-3M.

### 2.2. Disease Assessment of Wheat Powdery Mildew

**Figure 2:**
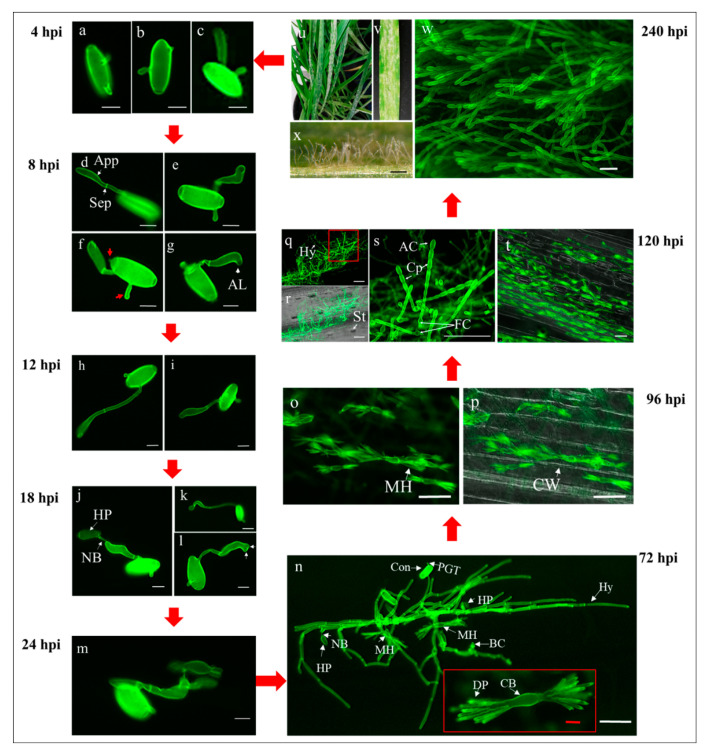
Disease cycle of *Bgt*#70 on leaf segments of LDN (susceptible parent). All samples were stained with WGA prior to observation under fluorescence microscope; (**a–c**) germination of *Bgt* conidia at 4 hpi; (**d–g**) initial stages of appressorial germ tube formation at 8 hpi; (**h,i**) advanced stages of appressorial germ tubes formation at 12 hpi; (**j–l**) initial stages of penetration and invasion of *Bgt* into wheat cells at 18 hpi; (**m**) development of mature haustoria at 24 hpi; (**n**) formation of *Bgt* colonies at 72 dpi. A high-resolution close-up image of the mature feeding structure of *Bgt* with central body and digitate processes (finger-like projections) is shown in the red box; (**o,p**) feeding structures of *Bgt* at 96 hpi (4 dpi). *Bgt* haustorium within the invaded plant host cell under fluorescence microscopy (**o**) and superimposition of fluorescence and bright field images with visible multiple haustoria with multi-digits in individual epidermal cells (**p**); (**q–t**) images of massive colonization and reproduction of *Bgt* on wheat leaves at 120 hpi (5 dpi) observed under fluorescence microscopy (**q,s,t**) and superimposed on a bright field images; (**r**) colonies with numerous conidiophores and haustoria are shown. The high-resolution close-up image is derived from the red box in q; (**u–x**) observation of *Bgt* symptoms and conidiophores developed at 240 hpi (10 dpi). Powdery mildew symptoms on intact leaves (**u**) and leaf segments (**v**), observation under fluorescence microscopy of *Bgt* conidiophores, aerial hyphae under light microscopy. AL: apical lobe; App: appressorium; AC: apical conidium; CB: central body; Con: conidium; Cp: conidiophores; CW: cell wall; DP: digitate processes (finger-like projections); BC: bulbous conidiophore; MH: mature haustorium; HP: haustorial primordium; Hy: hyphae; NB: neckband; Sep: septum; St: stomata. Scale bars: (**a–m**) and (**n**) red, 10 µm; (**o–t**) and (**n**) white, 50 µm; (**w**), 100 µm; (**x**), 250 µm.

The first signs of *Bgt* colonization were observed under the microscope on the susceptible LDN at 3 days post inoculation (dpi) and small *Bgt* colonies became visible to the naked eye at 4 dpi (Figure 1a and Figure 2n–p). The disease development progressed rapidly thereafter, and at 7 dpi the plants were heavily infected with massive powdery mildew colonies. Moreover, the mycelium color changed from white to gray-brown at 7 dpi. The aerial hyphae could be clearly observed under both light and fluorescence microscopy at 5 dpi (Figure 1a,b). In contrast, the resistant G305-3M leaves remained healthy and green, with a slight reddish discoloration most likely associated with the defense response (Figure 1a). The homozygous resistant F_6_ RIL CHR36 showed the same response to inoculation with *Bgt* as G305-3M, while the susceptible RIL CHR32 showed the same disease symptoms as LDN at 10 dpi (Figure 1c).

### 2.3. Microscopy of Bgt-Wheat Interactions within Infected Leaves

#### 2.3.1. Asexual Life Cycle of *Bgt* on Susceptible LDN Leaf Segments

**Figure 3:**
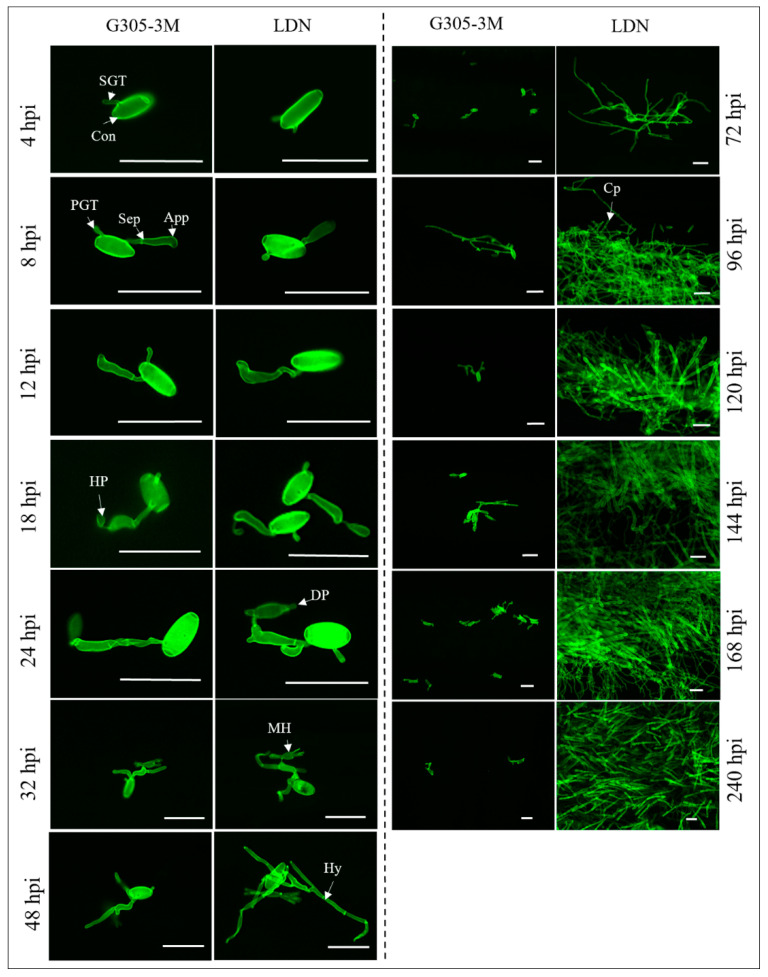
Microscopy of *Bgt*-wheat interactions on infected leaves in resistant versus susceptible lines. Segments of G305-3M and LDN leaves were observed under fluorescence microscopy after staining with wheat germ agglutinin (WGA) at 13 time points post inoculation. Scale bar is 50 μm. App: appressorium; Cp: conidiophore; Con: conidium; DP: digitate processes (finger-like projections); HP: haustorial primordium; Hy: hyphae; MH: mature haustorium; PGT: primary germ tube; Sep: septum; SGT: secondary germ tube.

In order to characterize the differences between compatible (susceptible) and incompatible (resistant) interactions, the asexual disease cycle of *Bgt* on leaf segments of LDN seedlings was followed along thirteen successive time points (4–240 h post inoculation (hpi); Figure 2 and Figure 3) through fluorescence microscopy observations.

At 4 hpi, the germinating conidial spores formed two types of germ tubes, a primary germ tube and a secondary germ tube. The germ tubes were mostly simple, straight, ended with rounded apices and were usually shorter in length than the conidial spores, and their positions relative to the center of conidia were terminal, subterminal or lateral (Figure 2a–c). Moreover, germ tubes emerging from a single conidium did not show a uniform pattern of position and length (Figure 2a–c).

At 8 hpi, the elongated secondary germ tubes began to differentiate into swollen appressorial germ tubes, separated by septa. Some of the appressorial germ tubes were longer than the conidia (Figure 2). As shown in Figure 2d–g, the appressoria were not uniform, exhibiting variable shapes and orientation relative to the central axis of the germinating conidia. Several examples are presented here: (i) a concomitantly straight appressorial germ tube and a short primary germ tube at the opposite terminal position (Figure 2d); (ii) a hooked appressorial germ tube and a short primary germ tube at the same terminal position (Figure 2e); (iii) two short primary germ tubes (red arrows) in addition to secondary appressorial germ tubes (Figure 2f). Hooked appressoria with an apical lobe that start to penetrate the epidermal cells can be observed (Figure 2g).

At 12 hpi, most of the germinating conidia formed appressoria with typical apical hooks and characteristic septa that separate the differentiated appressoria cells from the conidia (Figure 2h,i). Most of the appressorial germ tubes were longer than the conidia. Within the following six hours, penetration pegs were formed and produced bulb-like haustorial primordia inside the epidermal host cells (Figure 2j–l). Haustorial primordia at the end of the swollen (Figure 2j) and elongated (Figure 2k) appressoria, with primary germ tubes at different positions of the conidium were observed. Elongated and branched appressoria were also observed (Figure 2l, branches marked with arrows).

Subsequently, the typical digitate processes (finger-like projections) of haustoria were visible at 24 hpi. The formation of secondary hyphae was observed branching from the established appressorium (Figure 2m). At 72 hpi, extensive hyphal growth and repeated penetration from hyphal appressoria resulted in the formation of further digitate haustoria, and establishment of bulbous conidiophores on host epidermal cells (Figure 2n). The mycelial hyphae developed and proliferated on the plant leaf surface at 96 hpi (Figure 1) were accompanied and supported by massive invasion into the epidermal cell layer and formation of numerous haustoria which are feeding the pathogen colonies via multiple digit structures (Figure 2o,p). At this stage, the first batch of conidiophores already began to appear (Figure 3, LDN 96 hpi) and the first signs of disease can be visualized macroscopically (Figure 1, LDN 4 dpi).

At 120 hpi, massive airborne conidiophores were produced; a surge of haustoria in epidermal cells were accompanied by well-developed colonies (Figure 2q–t). From 120 to 240 hpi there was a sharp increase in sporulation. Colonies were well developed in the susceptible LDN, with many outward projections of chains of conidia (Figure 2u–w). Interestingly, the development of hypha was consistent with the direction of the vein extension and the tightly crisscrossed hyphae had close adhesion with the epidermal cells. Moreover, the distribution of haustoria in epidermal cells suggested that hyphae were growing mainly along the vertical direction of the leaves (Figure 2t). When the young conidia were released from the conidiophores, they initiated a second cycle of inoculation and infection of the host plants, indicating the start of a new disease cycle.

#### 2.3.2. Comparison of Fungal Development on Leaves of Susceptible LDN and Resistant G305-3M

A comparison of fungal development on susceptible LDN and resistant G305-3M young leaves revealed near identical developmental processes during the initial stages of infection, from the point of landing of conidia on the host leaves until the formation of haustorium primordia (0–24 hpi), while striking differences in the extent of *Bgt* development from 32 to 240 hpi were observed for LDN (Figure 3). In the LDN leaves, *Bgt* hyphae were abundant on the leaf surface and penetrated freely the epidermal cells to form mature haustoria within these cells (Figure 3). In contrast, the growth of *Bgt* on the G305-3M leaves was very limited. Only a few germinated conidia with secondary hyphae could be observed, while the development of most of the germinating conidia was blocked at the haustoria primordium stage (Figure 3).

The resistant RIL CHR36 showed arrest of pathogen development at the haustorial primordia, the same pattern as G305-3M (Supplementary Figure S1, 4 & 10 dpi), with only a few conidia that developed mature haustoria and secondary hyphae, while the susceptible RIL CHR32 presented the same infection pattern as LDN, with well-developed haustoria at 4 dpi, and massive development of conidiophores at 10 dpi (Supplementary Figure S1).

Interestingly, we observed that appressoria of the conidia located near stomata avoided penetrating the stomatal guard cells in both resistant G305-3M and susceptible LDN (Supplementary Figure S2). Even in those cases where spores landed on the stomatal guard cells, the appressoria were extended and avoided guard cell penetration (Supplementary Figures S1b and S2c). Moreover, although multiple mature haustoria formed in one single epidermal cell in LDN at 4 dpi, no mature haustoria were observed in stomatal guard cells (Supplementary Figure S2d).

#### 2.3.3. The Early Stages of *Bgt*-Wheat Incompatible Interaction in Leaves of the Resistant Line G305-3M 

**Figure 4:**
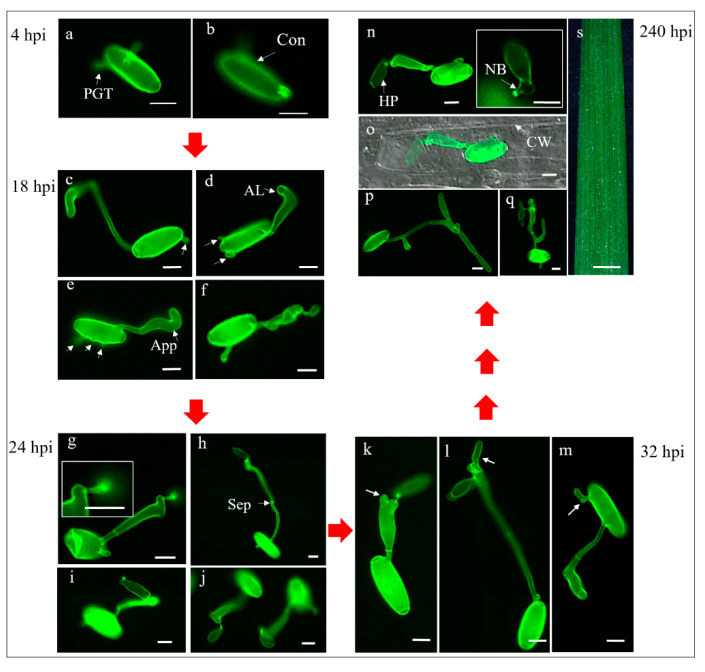
The early stages of *Bgt*-G305-3M incompatible interaction observed after inoculation of resistant leaf segments with isolate *Bgt*#70. All samples were observed under fluorescence microscopy after staining with WGA; (**a,b**) germination of *Bgt* conidia at 4 hpi; (**c–f**) early stages of penetration into host cells at 18 hpi. Arrest of penetration of *Bgt* into host cells at 24 hpi (**g–j**) and 32 hpi (**k–m**); (**n,o,s**) arrest of development of *Bgt* feeding structures within host cells at 240 hpi; (**p,q**) the development of *Bgt* within host cells at 240 hpi. Scale bar is 10 μm in (**a–q**), 1 mm in (**s**). AL: apical lobe; App: appressorium; Con: conidium; CW: cell wall; HP: haustorial primordium; NB: neckband; Sep: septum.

The early stages of *Bgt-*wheat interactions were examined in leaves of the resistant line G305-3M (4–240 hpi) using fluorescence microscopy after staining with WGA (Figure 3 and Figure 4).

The development of primary germ tubes of the germinating *Bgt* conidia observed at 4–12 hpi showed the same pattern of development on both the resistant G305-3M and the susceptible LDN leaves (Figure 2, Figure 3 and Figure 4). At 18 hpi the conidia germinating on G305-3M leaves already produced 1–3 primary germ tubes and fully developed hooked appressoria (Figure 4c, one primary germ tube; d, two; e, three; marked with white arrows). In some cases, a premature haustorial primordium was observed extending from the mature appressorium (Figure 3 and Figure 4f).

In most cases, haustorial primordia connected to the swollen (Figure 4g) or elongated (Figure 4h) appressoria via neckbands could be observed at 24 hpi. Bulb-like haustorial primordia connected to appressoria via neckbands were very common at this stage (Figure 4j), whereas preliminary formation of digitate processes were observed only in rare cases (Figure 4i). At 32 hpi, primary germ tubes with branched appressoria (white arrows) and haustorial primordia were commonly observed (Figure 4k,l), while branched primary germ tubes (white arrow) were observed only in very rare cases (Figure 4m).

From 32 hpi to 240 hpi, for most conidia, there were no further changes in *Bgt* haustoria development within the invaded host plant cells, as observed in fluorescent images (Figure 4n) superimposed on bright field images (Figure 4o); the development of haustoria in G305-3M were arrested at haustorial primordia stage (Figure 4n,o) and no digitate processes were formed. Nevertheless, in a few cases some conidia were able to form haustoria with digitate processes and relatively short secondary hyphae (Figure 4p,q).

Taken together these results indicate that the resistance gene *PmG3M* activates a post-haustorial resistance mechanism, which leads to the arrest of the fungal feeding structures and termination of fungal colony growth on the leaf surface probably due to lack of nutrients.

### 2.4. The Accumulation of Fungal Biomass within Susceptible (LDN and CHR32) Versus Resistant (G305-3M and CHR36) Wheat Lines after Inoculation with Powdery Mildew

**Figure 5:**
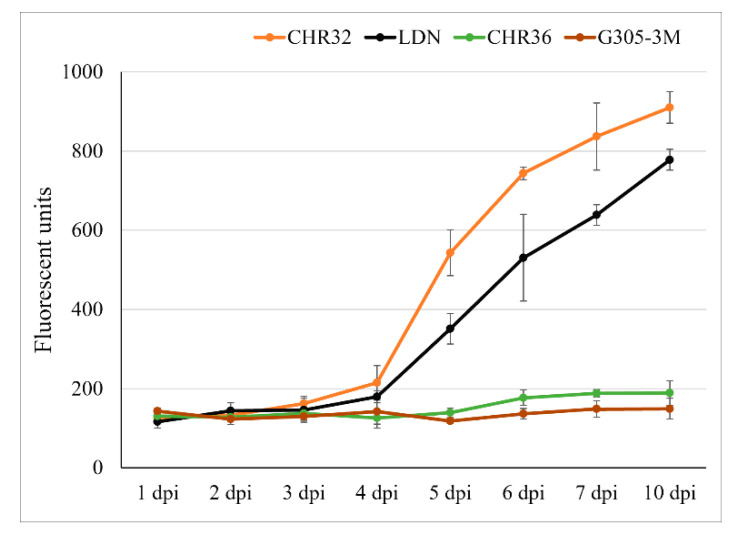
Accumulation of fungal biomass (chitin) within leaf tissues of inoculated wheat lines (LDN and CHR32) susceptible to *Bgt*, as compared with resistant lines (G305-3M and CHR36) from 1 to 10 dpi after inoculation with *Bgt*#70. Error bars denote standard deviation (s.d.) based on five biologic replicates. dpi: days after inoculation. Duncan’s multiple range test is described in Supplementary Figure S3.

Fungal biomass was assessed by measurement of amount of chitin present in fungal cell walls, but absent in plant cell walls. Within leaf tissues of susceptible CHR32 and LDN it increased sharply from 4 to 10 dpi and the relative quantification of fungal development and colonization fit a typical standard logistic population growth model (LDN: *p*  <  10^−10^; CHR32: *p*  <  10^−8^) (Figure 5). Moreover, the susceptible CHR32 displayed a significantly higher increase in fungal biomass than LDN at the same time frame (5–10 dpi, Figure 5 and Supplementary Figure S3). In contrast, fungal biomass accumulation in the leaves of the resistant lines G305-3M and CHR36 was very low and did not change significantly over the sampling period (1–10 dpi, Figure 5 and Supplementary Figure S3).

A comparison of fungal biomass accumulation between the four tested lines revealed no significant differences between the resistant (G305-3M and CHR36) and susceptible (LDN and CHR32) lines during the first three time points (Figure 5 and Supplementary Figure S3). At 4 dpi, significant differences (*p* < 0.01) were observed between the susceptible CHR32 and the resistant lines (G305-3M and CHR36). At the same time point, LDN had 42.2% and 26.2% higher average fungal biomass accumulation compared to CHR36 and G305-3M, but these differences were not significant (Supplementary Figure S3). Significant differences between the resistant and susceptible lines were detected at 5 to 10 dpi (Supplementary Figure S3), at about the time the mycelium became visible under macroscopic observation on CHR32 and LDN (Figure 1). Microscopic examination revealed that during this period, germination of *Bgt* conidia in these two susceptible lines was followed by the elongation and secondary penetration of hyphae and generation of multiple haustoria in epidermal host cells, leading to massive colonization of epidermal cells, as compared to the arrest of fungal development in the resistant lines (Figure 2, Figure 3 and Figure 4).

### 2.5. Epidermal Cell Responses to Bgt Inoculation in Resistant Versus Susceptible Host Plants

#### 2.5.1. Callose Deposition in Resistant Versus Susceptible Host Plants after *Bgt* Inoculation

**Figure 6:**
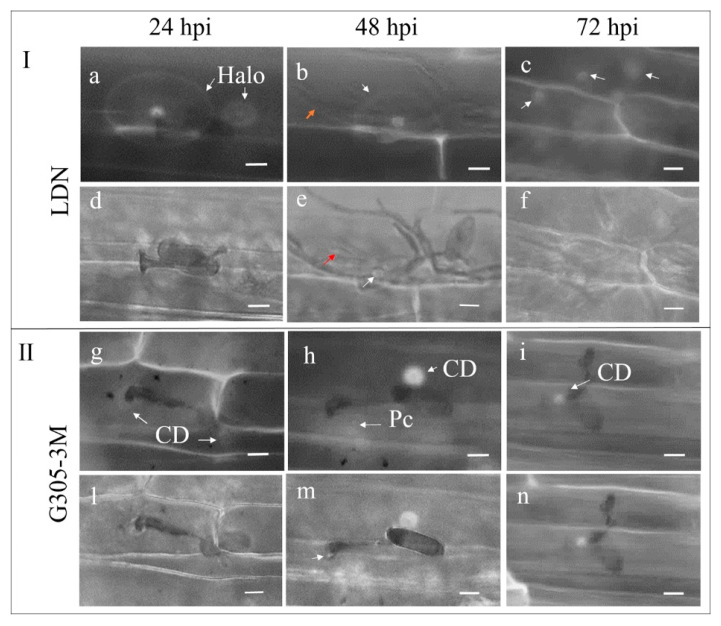
Fluorescence microscopy observation of callose deposition in young leaves of resistant G305-3M and susceptible LDN at 24, 48 and 72 hpi after *Bgt*#70 inoculation. In both panels, G305-3M and LDN, the upper micrographs (**a–c,g–i**) were obtained under fluorescence microscopy after aniline blue staining, while the lower micrographs (**d–f,l–n**) show callose deposition observed after superimposition of fluorescence and bright field microscope images of inoculated leaf samples stained with aniline blue. CD: callose deposition; Pc: patch-like callose deposition. Scale bar is 50 μm.

The attempts of *Bgt* hyphae to penetrate into plant cells were often associated with the deposition of wall papillae (Figure 6). In the susceptible LDN, we observed callose at 24 hpi under fluorescence microscopy as an aperture-like halo, with a bright core encasing both the penetration sites of appressorium and primary germ tube. The field of callose deposition that formed under the appressorium penetration site was much larger and brighter than that observed under the penetration site of primary germ tube (Figure 6a). The localization of the oval halos to the attacked epidermal cell beneath the appressorium and primary germ tube was validated by superimposition of fluorescence and bright field microscopy images after aniline blue staining (Figure 6a,d). In the resistant G305-3M, a field of callose deposition can be observed at 24 hpi under the appressorium and a smaller one at the primary germ tube penetration sites (Figure 6g). The localization of the hemispherical papilla with auto fluorescent material directly beneath the *Bgt* appressorium and a slight papilla formed at the unsuccessful penetration site of primary germ tubes were observed by superimposition of fluorescent and bright field microscopy images after aniline blue staining (Figure 6g,l).

The halo aperture-like callose deposition (white arrow) with a dot-like core in the susceptible LDN with elongated secondary hyphae (orange arrow) was observed at 48 hpi (Figure 6b), while the digitate processes (red arrow) of mature haustorium and papillae (white arrow) at the penetrated site were found under the superimposition of fluorescence and bright field observation (Figure 6e). A bright dot-like callose deposition can be seen at 48 hpi in G305-3M as a response to the attack by primary germ tube, while a patch-like formation of callose deposition emerged beneath the appressorium (Figure 6h,m). The localization of the smaller size papilla (white arrow) at the primary germ tube penetration site and a patch-like callose deposition beneath the appressorium was visible under the superimposition of fluorescence and bright field microscopy images (Figure 6m).

At 72 hpi large *Bgt* colonies were already formed in LDN and many dot-like callose depositions induced by hyphal appressoria were observed beneath them (Figure 6c,f). However, only smaller dot-like callose depositions at the primary germ tube penetration sites were observed in the resistant G305-3M (Figure 6i,n).

#### 2.5.2. H_2_O_2_ Accumulation and Plant Cell Death in Epidermal Cells in Response to *Bgt* Inoculation

**Figure 7:**
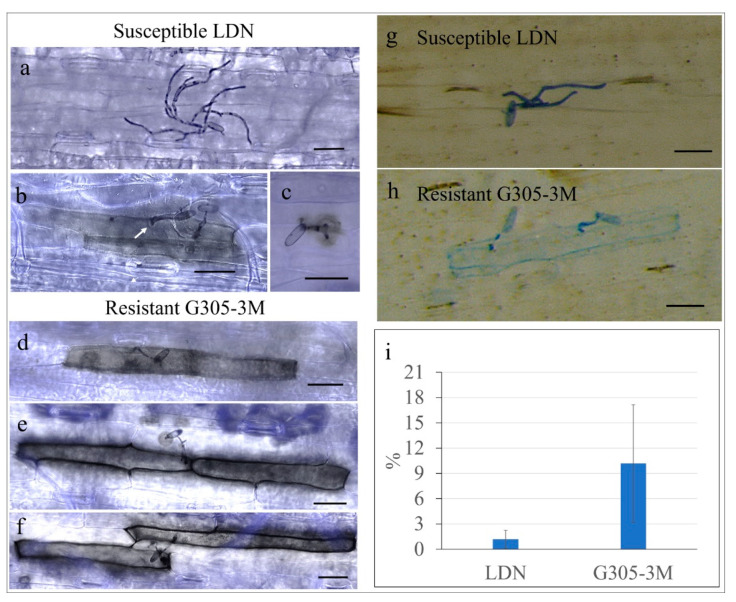
H_2_O_2_ accumulation and programmed cell death in young leaves of resistant G305-3M and susceptible LDN at 2 dpi inoculated with *Bgt*#70. The leaf samples in (**a–f**) were stained with DAB-aniline blue, while those in (**g,h**) were stained with trypan blue. All micrographs were observed under a light microscope; (**a–c**) H_2_O_2_ accumulation and epidermal cell response of susceptible LDN leaves upon *Bgt* penetration; (**d–f**) H_2_O_2_ accumulated in the epidermal cells of resistant G305-3M leaves upon *Bgt* penetration. Scale bar is 50 μm. (**i**) The percentage of germinated *Bgt* conidia which showed H_2_O_2_ accumulation in the resistant G305-3M and susceptible LDN leaf segments. Error bars denote standard deviation based on nine biologic repeats.

H_2_O_2_ accumulation was monitored during the penetration stages of *Bgt* hyphae into epidermis plant cells (1–3 dpi, Supplementary Figure S4). In the susceptible LDN, most *Bgt* conidia developed well with the elongated hyphae with no H_2_O_2_ accumulation at 2 dpi (Figure 7a). However, a few *Bgt* conidia (1.2% of germinated spores) had some H_2_O_2_ accumulation within the penetrated plant cells, as indicated by dark-brown staining from DAB polymerization (Figure 7b,c,i). In these rare cases, papillae (white arrow) could be observed in the same epidermal cell, but neither a haustorium nor secondary hypha were formed (Figure 7b,c). In the resistant G305-3M, 10.2% of the germinated *Bgt* conidia showed substantial accumulation of H_2_O_2_ in the penetrated epidermal cells and cell walls (Figure 7i). As shown in Figure 7d, the penetration of a host cell by a single *Bgt* conidium resulted in the accumulation of high amount of H_2_O_2_ within a single host cell. In some cases, when the *Bgt* penetration occurred close to the border between two neighboring epidermal cells, both showed high accumulation of H_2_O_2_ (Figure 7e). We also observed a small amount of H_2_O_2_ accumulation, which looks like a brownish hallo, occurring at the attack site of the primary germ tube of the same conidium (Figure 7e). A similar case is shown in Figure 7f in which a single *Bgt* conidium caused high H_2_O_2_ accumulation on two adjacent epidermal cells by forming a primary germ tube on one of them and appressorium on another. The two adjacent cells were surrounded by alive epidermal cells, which showed no accumulation of H_2_O_2_. Moreover, neither papillae nor digitated haustorium were seen in these invaded cells. In most cases, *Bgt* developed normally in the susceptible LDN and the infected cells were not stained with trypan blue indicating that they are indeed alive (Figure 7g). In contrast, in most cases, the *Bgt* invaded host cell of the resistant line G305-3M were stained with trypan blue, indicating that these cells were dead, likely as a result of induced programed cell death (Figure 7h).

## 3. Discussion

During the last two decades, advanced technologies that allow researchers to explore various aspects of host-pathogen interactions at the cellular and molecular levels were developed and used to clone novel plant disease resistance genes and discover their roles in the defense response against pathogens (e.g., [14,15]). Pathogen effector proteins were also studied extensively to expound upon resistance-effector interactions and plant NLR-triggered immunity (e.g., [16,17]), as well as the involvement of plant hormones and ROS signaling in plant defense mechanisms (e.g., [8,18]). Nevertheless, microscopic observations throughout the infection process remain the classical approach to study the histopathology of defense responses in host–pathogen interactions, as we used here for histological characterization of the *PmG3M-*mediated resistance to wheat powdery mildew.

In the current study, we used wheat germ agglutinin staining and advanced fluorescence microscopy imaging technologies to study the penetration process of germinating *Bgt* spores into wheat epidermal cells and the development of fungal feeding structures within the cells of a susceptible host during the asexual life cycle of *Bgt*. Our results confirmed previous reports stating that the asexual life cycle of powdery mildew proceeds in a strictly programmed manner and is highly synchronous [5]. Pre-penetration of conidia occurred upon landing on the host surface, indicated by the formation of a short aseptate primary germ tube, whose functions are to rapidly attach the fungal germling to the host surface, gain access to host water during pre-penetration and recognize characteristics of the contact surface [5]. Our results corroborate the report of Jankovics et al. [6], showing that more than one primary germ tube can germinate from a single conidium, and those primary germ tubes are distributed randomly along the conidium without uniform rules. The fully developed haustoria with numerous finger-like digitate processes were formed to increase the contact surface area with host cells in order to increase the absorption of host nutrients during the invasion stage. Subsequently, well-developed colonies were established on the susceptible host.

*Bgh* infection was previously shown to impair stomatal behavior in both susceptible and resistant barley genotypes after penetration of the pathogen into stomata neighboring cells [19]. However, this disruption was associated with the interaction with epidermis host cells and was not attributed to penetration into stomata guard cells. In the current study, we show evidence that *Bgt* appressoria tend to avoid penetration into stomata guard cells (Supplementary Figure S2), a finding supported by Prat et al. [19]. Therefore, we assume that powdery mildew fungi are recognizing and specifically targeting epidermal plant cells, rather than stomata guard cells. However, confirmation of such assumptions requires further experimentation which is beyond the scope of current study.

The current histopathology study is part of a long-term project aiming to clone and characterize powdery mildew and stripe rust resistance genes derived from wild emmer wheat by using the map-based cloning approach, as we recently demonstrated for the cloning of *Yr15* [14,20]. Previously, we localized *PmG3M* to chromosome 6B using the G305-3M × LDN mapping population [12]. To confirm the single-dominant gene inheritance of resistance in *PmG3M*, we tested the segregation ratio in G305-3M × LDN mapping population (265 F_2_ plants) and showed that only one resistance gene is segregating there (3R:1S). In addition, we have selected to identify two RILs that represent reciprocal-like recombination events between marker *stm583* and *PmG3M*, and therefore carry alternative parental alleles of *PmG3M*. The homozygous resistant (CHR36) and homozygote susceptible (CHR32) RILs expressed resistance/susceptibility responses as their parents, therefore validating that the resistance response is mediated by *PmG3M*.

Furthermore, we demonstrated that the *Bgt* asexual life cycle was completed on leaves of LDN and CHR32, but not on G305-3M and CHR36. In the resistant lines, the penetration process, haustorium development and further colonization of host cells were stopped at the haustorium primordia stage. In only a few cases digitate processes could be observed, leading to the formation of short secondary hyphae, while in most cases bulb-like haustoria without any typical finger-like lobes were formed, as a result of the *PmG3M*-mediated resistance mechanism. Numerous studies have demonstrated that mature haustoria are crucial for successful infection and colonization of host tissue by biotroph pathogen, since they allow absorption of nutrients from the host cells and delivery of a remarkable diversity of fungal effectors that manipulate host immunity and metabolism [21,22]. In *Bgh* alone, almost 500 genes encoding for candidate secreted effector proteins (CSEPs) were identified and a large part of them showed a significantly higher relative expression in haustorial tissue, than the epiphytic tissue [23]. Moreover, most CSEPs (79%) in *Bgh* genome are preferentially expressed in haustoria, which may indicate that they have specific functions in biotrophic pathogenesis [24]. Nevertheless, fungal effectors also play an important role in incompatible interactions by activating the resistance mechanism of host plants that carry specific receptors and recognize these effectors in a gene-for-gene manner (e.g., [25]). The *AvrPm3-Pm3* effector-NLR interactions were shown to control both race-specific resistance and host-specificity of cereal mildews on wheat [26]. Therefore, the wheat-*Bgt* pathosystem is providing an excellent example for avirulence factors that are highly expressed at the stage of haustorium formation and recognized by *Pm* resistance genes, therefore demonstrating the importance of haustorial formation for the plant–pathogen interactions [16]. In the case of *PmG3M* further work is under way to clone the gene and identify its role in activating the resistance response.

Based on the accumulated results obtained using different genetic backgrounds, we can clearly demonstrate that the powdery mildew resistance mediated by *PmG3M* includes a post-haustorial resistance mechanism, which leads to the arrest of the fungal feeding structures and cessation of fungal colony growth on the leaf surface due to lack of nutrients. This resistance mechanism is different from the *mlo* powdery mildew resistance gene [27], which effectively arrest fungal pathogenesis at very early stages of infection prior to formation of haustoria and secondary hyphae (a pre-haustorial resistance mechanism). Although, *Bgh* spores make an attempt to penetrate epidermal host cells, they fail—and thus the primary formation of haustorium is prevented, sporulation does not happen and the pathogen cannot finalize its asexual life cycle [27,28]. Recent studies have demonstrated that *mlo*-mediated resistance can be effectively induced in broad range of monocotyledonous and dicotyledonous plant species, including wheat [28]. Similarly, during melon-powdery mildew (*Podosphaera fusca*) interactions, the *P. fusca* hyphae developed abundantly in the susceptible cultivar ‘Rochet’ with a large number of haustorial formations, while fungal growth is arrested at the first germ tube stage, prior to haustoria formation in the resistant line ‘PMR-6’ [29]. In the compatible and incompatible *Arabidopsis*-powdery mildew interactions, haustoria formation was prevented in resistant transgenic lines during both types of powdery mildew inoculation (*Golovinomyces cichoracearum* and *Bgh*), but developed well in susceptible lines [30]. Furthermore, the powdery mildew resistance gene *Mla* activated post-haustoria resistance mechanism that restricted *B. graminis* growth [31]. Although the directions and purposes of various host-powdery mildew studies were different, they all demonstrate that preventing haustoria formation is a conserved mechanism to arrest fungal growth and to control disease development.

Callose-containing cell-wall appositions are effective barriers that are induced at the sites of attack during the relatively early stages of pathogen invasion. However, previous studies on the function of callose deposition in host–pathogen interactions have been controversial. The work by Consonni et al. [32] demonstrated that callose deposition was irrelevant for plant resistance to adapted biotrophic powdery mildews. In contrast, Ellinger et al. [30] found that penetration resistance was important to the resistance response when callose was deposited early during the infection process and that inhibition of callose deposition was correlated with increased susceptibility of *Arabidopsis* to fungal pathogens [33]. Chowdhury et al. [34] then provided evidence that callose deposition positively contributes to the barley fungal penetration resistance mechanism against *Bgh*. Previous reports have demonstrated that callose deposition is part of the basal defense responses of plant immunity against pathogens typically triggered by conserved pathogen-associated molecular patterns (PAMPs) (reviewed by Claverie et al. [35]). In our study, we have shown that callose deposition occurred at the fungal penetration sites, in both the resistant (G305-3M) and susceptible (LDN) genotypes, suggesting that although some basal defense responses were activated, they were not effective against virulent *Bgt,* which successfully penetrated into host cells of both genotypes. Fungal development was arrested only in G305-3M and CHR36 by a post-haustorial resistance mechanism mediated by *PmG3M.*

Powdery mildew is an obligate parasite which can successfully grow and multiply only on living host plant cells and therefore, programmed cell death is a major component of the resistance response against this destructive disease (reviewed by Liu et al. [36]). Hydrogen peroxide (H_2_O_2_) accumulation and cell death were shown to be induced after inoculation with *Bgt* spores in resistant wheat lines that harbor *Pm60* [37]. In the current study, epidermal cells and cell walls of G305-3M showed strong H_2_O_2_ accumulation after *Bgt* inoculation, likely leading to programmed cell death of the infected epidermal cells, as evident from staining of the *Bgt*-penetrated cells with trypan-blue, a dye that is used for measuring cell viability. Moreover, H_2_O_2_ accumulation could sometimes be observed in two adjacent epidermal cells, infected by a single conidium, and it seems that both the partial penetration of one epidermal cell during attachment of the *Bgt* conidium via the primary germ tube and the full penetration of the second host cell via the formed appressorium, penetration peg and premature haustorium resulted in H_2_O_2_ accumulation. The relatively low percentage of infected epidermal cells that showed extensive H_2_O_2_ accumulation can be explained by the instability of ROS and timing of staining. As noted by Mittler [38], H_2_O_2_ accumulation is a dynamic process, and H_2_O_2_ can be degraded very fast, because ROS scavenging mechanisms are usually induced rapidly [38]. The trypan blue staining of infected G305-3M epidermal cells indicates that they are not viable [39], probably due to programed cell death induced by *PmG3M*. In contrast, LDN infected cells did not stain with trypan blue, indicating that they are alive. These results demonstrate that the notable defense responses, such as H_2_O_2_ accumulation and programmed cell death in epidermal cells play important role in the *PmG3M*-mediated wide-spectrum resistance.

Our results also showed that CHR32 had significantly higher accumulation of fungal biomass than the parental line LDN from 5 dpi to 10 dpi. Together with slight H_2_O_2_ accumulation in *Bgt* infected LDN, it may suggest that some partial resistance mechanisms are defending against powdery mildew in LDN background. Ben-David et al. [40] provided evidence that LDN possessed resistance responses to some of the tested *Bgt* isolates and LDN contributed to four isolate-specific quantitative resistance loci to improved powdery mildew quantitative resistance to *Bgt* (lower density of fungal colonies on the leaves). However, it seems that some susceptible RILs (e.g., CHR32) lost some of the defense responses harbored by LDN, leaving them even more susceptible to powdery mildew, as a result of transgressive segregation.

In summary, we have shown that haustoria were abnormally shaped in the resistant G305-3M leaves, accompanied by a series of epidermal cell reactions that apparently suppressed the multiplication and colonization of the fungus, including callose deposition, H_2_O_2_ accumulation, hypersensitive response and programmed cell death. A post-haustorial resistance mechanism was also observed that arrested the development of fungal feeding structures and protected the epidermal cells from an extensive fungal invasion. Further work is underway aiming to clone *PmG3M* and elucidate the molecular mechanism of resistance conferred by this gene. This will open the door to developing novel strategies for engineering enhanced protection against powdery mildew pathogens.

## 4. Materials and Methods

### 4.1. Plant Materials and Bgt Isolate

The WEW accession G305-3M, donor of *PmG3M*, and *T. durum* cv. LDN parental lines were used to produce homozygous F_6_ recombinant inbred line (RIL) population by the single seed decent procedure, as previously described [11,12]. In the current study, we used one resistant RIL (CHR36) and one susceptible RIL (CHR32), derived from the same cross that carried alternative chromosome segments with alternative alleles of *PmG3M* (Supplementary Table S1). The recombination events carried by these RILs in the *PmG3M* chromosome region were validated by molecular markers *stm583* and *edm149* ([11]; Supplementary Tables S1 and S2). Furthermore, we have generated an additional population (G305-3M × LDN) of 265 F_2_ plants and phenotyped them to validate the presence of a single *Pm* resistance gene (Supplementary Table S3).

*Bgt* isolate #70, used in the current study, is highly virulent on many known powdery mildew resistance genes (e.g., *Pm1a*, *Pm1b*, *Pm2*, *Pm3a*, *Pm3b*, *Pm3c*, *Pm3d*, *Pm5a*, *Pm5b*, *Pm6*, *Pm7*, *Pm17*, *Pm22* and *Pm1* + *Pm2* + *Pm9*; [41]). G305-3M and CHR36 are highly resistant to infection with *Bgt* isolate #70 (IT = 0), while LDN and CHR32 are susceptible (IT = 4) [11,12].

### 4.2. Bgt Infection and Disease Assessment

All plant materials were tested at the two-leaf stage and the results were recorded at 7 and 10 dpi. Conditions of inoculation, incubation and disease assessment were as described by Hsam & Zeller [42]. In brief, the first leaf of each plant at the seedling stage was cut into three segments and cultured on 8 g/L agar containing 50 mg/L benzimidazole (Sigma-Aldrich, St. Louis, MO, USA) in square petri dishes. After even inoculation with powdery mildew fresh spores, the samples were incubated at 20–22 °C with a 16-hour photoperiod. The reactions to *Bgt* inoculation were examined visually, with the infection type (IT) recorded based on a scale of 0 to 4 [12].

### 4.3. Microscopic Observations of Wheat-Bgt Interactions within Infected Leaves

Fluorescence microscopy of *Bgt* structures was performed using wheat germ agglutinin (WGA; a lectin that binds specifically to β (1→4)-N-acetyl-D-glucosamine, i.e., chitin) conjugated with a fluorescent dye, according to Dawson et al. [43] and Klymiuk et al. [14,20], with slight modifications. Leaf segments (primary leaf, 5 cm long) were sampled at 4, 8, 12, 18, 24, 32, 48, 72, 96, 120, 144, 168 and 240 h post inoculation (hpi) with *Bgt* spores. Harvested leaf segments were incubated in 1 M KOH at 37 °C for 24 h, neutralized in 50 mM Tris, pH 7.0, stained with 20 µg/mL solution of WGA conjugated to fluorophore Alexa 488 (L4895-2MG; Sigma-Aldrich), then washed and mounted on microscope slides with fluorescence antifade mounting medium (Vectashield, Vector Laboratories, Burlingame, CA, USA), covered and sealed with cover glass. The sealed samples can be stored at 4 °C in the dark over a month. An inverted fluorescence microscope, Leica DMi8 (Leica Microsystems, Wetzlar, Germany), fitted with a filter cube for the FITC excitation range (Ex: 460–500; Dc: 505; Em: 512–542) and a FLUO regime, was used to observe the WGA-stained fungal structures.

Fluorescence microscopy of callose deposition was performed using aniline blue (Sigma-Aldrich) to demonstrate the presence of β-1,3-glucan in the plant cell walls penetrated by the fungal pathogen, according to Lyngkjær & Carver [44]. In brief, the leaf segments (primary leaf, 5 cm long) were placed, with the inoculated surfaces up, onto Whatman #1 paper moistened with ethanol and acetic acid (3:1, *v/v*) until completely bleached (approximately 16–20 h). Then the samples were transferred to filter paper moistened with the clearing solution lactoglycerol (lactic acid: glycerol: water (1:1:1, *v/v*/v)) for 3 h. Finally, fungal structures were stained for 30 min by carefully pipetting a few droplets of 0.05% (*w/v*) aniline blue in acetic acid: glycerol: water (1:1:1, *v/v*/*v*) onto the inoculated surface. The fluorescence microscopy was performed on an inverted fluorescence microscope, Leica DMi8 using the 4′,6-diamidino-2-phenylindole (DAPI) filter set (Ex: 325–375 nm; Em: 435–485 nm). During our microscopic observations, we could observe obvious dot-like callose deposition within blue backgrounds. However, in order to improve the contrast between callose depositions and background, we have changed the color saturation in the presented images.

To visualize hydrogen peroxide (H_2_O_2_) accumulation, 3,3’-diaminobenzidine (DAB) staining was performed on leaf segments inoculated with *Bgt* as described by Thordal-Christensen et al. [45]. The primary leaves inoculated with *Bgt*#70 were cut, placed in 1 mg/mL 3,3’-diaminobenzidine (DAB)-HCl at pH 3.8 (as a low pH is necessary to solubilize DAB) and incubated at 20 °C for 8 h prior to sampling. The leaf segments were then cleared in boiling ethanol (96%) for 15 min. H_2_O_2_ is visualized as a reddish-brown coloration using fluorescence microscope with a DAPI filter. For DAB observation, the images were recorded after superimposition of bright field and fluorescence after aniline blue-DAB staining. In our preliminary testing of H_2_O_2_ accumulation (Supplementary Figure S4), we could observe clear H_2_O_2_ accumulation in plant cells of G305-3M at 2 and 3 dpi. In previous studies it was shown that H_2_O_2_ accumulation was detected at 2 dpi [37]. Therefore, we have selected 2 dpi for a more detailed examination (Figure 7).

Since trypan blue stains dead plant cells and fungal hyphae [39], we used it to observe plant cell death after *Bgt* inoculation. This experiment was performed with inoculated samples at 2 dpi via a similar procedure as in the observation of callose deposition. After trypan blue (Sigma-Aldrich) staining, the samples were observed under a light microscope (Nikon, AZ100, Tokyo Met., Japan).

All microscopic observation experiments were repeated 2–3 times, with at least three biologic replicates each time.

### 4.4. Quantification of Fungal Biomass within Infected Leaves

The quantification of fungal biomass was based on the estimation of chitin content using chitin binding WGA conjugated to fluorophore fluorescent dye as described by Ayliffe et al. [46] and Klymiuk et al. [14] with slight modifications. After inoculation with *Bgt* isolate #70, leaf segments of the two parental lines (G305-3M and LDN) and the two RILs (CHR36 and CHR32), were collected 1–7 dpi and 10 dpi from different plants at each time point. Five biologic replicates were collected from each line at each time point and used for quantification of fungal biomass by chitin measurement. Leaf segments were weighed, cut into 3–4 cm pieces, placed in 15-mL Falcon tubes and covered with 5–6 mL of 1 M KOH containing 2–3 drops of the surfactant alkylaryl polyether alcohol (Spreader DX). Plant materials were autoclaved using a standard sterilization cycle (121 °C and 15 psi for 20 min) followed by quick washing with 15 mL of 50 mM Tris HCl, pH 7.0 and neutralization by a second wash with 15 mL of 50 mM Tris HCl, pH 7.0 for at least 15 min. Centrifugation for 5 min at 4700× *g*, was used during all steps to avoid losing fungal materials. To avoid destroying the fragile leaf tissues, the leaf segments were gently moved to a new falcon tube, then the remaining solution was spun down the fungal materials were collected at the bottom. Neutralized tissues and spun down materials were carefully transferred to 1.7 mL microcentrifuge tubes that contained 1 mL of 50 mM Tris HCl, pH 7.0 for each 200 mg of fresh leaf tissue, according to measurements conducted at harvest time. Leaf samples were then homogenized at 30 Hz for 2 min in a TissueLyser II (Qiagenza) and a 100 µL aliquot of each sample suspension was transferred to a 200-µL microcentrifuge tube that contained 10 µL of a 0.5 mg/mL solution of WGA conjugated to fluorophore Alexa 488 dissolved in water. Three technical replicates were used for each tissue sample. The ends of pipette tips were removed prior to all sample pipetting and homogenates were regularly agitated to ensure that a uniform sample was collected. The sample and stain solution were well mixed by repetitive pipetting and left to stand for 30 min at room temperature. Following staining, samples were centrifuged at 600× *g* for 3 min. The supernatant containing unbound stain was removed by pipetting and the pellet was resuspended in 200 μL of 50 mM Tris, pH 7.0. Samples were washed six times in 200 μL of 50 mM Tris (pH 7.0), resuspended in 100 μL of 50 mM Tris (pH 7.0) and transferred to black, 96-well microtiter trays for fluorometry. Fluorometric measurements were conducted using a SpectraMax M2e Microplate Reader (Molecular Devices, Sunnyvale, CA, USA) set with 485-nm excitation and 535-nm emission wavelengths, a 1.0 s measurement time and a cross pattern of well-scanning, yielding an average measurement per well.

In addition, to optimize the protocol for measurement of fungal biomass, the background fluorescence from the host plant was documented and taken into account when performing the measurements (Supplementary Figure S5).

### 4.5. Statistical Analysis

Chi-squared distribution analysis was carried out to determine the segregation ratio of *Bgt* phenotype responses of F_2_ plants from the G305-3M×LDN mapping population. The percentage of germinated *Bgt* conidia which resulted in H_2_O_2_ accumulation in the resistant G305-3M and susceptible LDN leaf segments were analyzed through SPSS (SPSS 16.0, SPSS, Inc; Chicago, IL, USA).

Statistical analyses of the accumulation of fungal biomass were determined by the following linearized formula to identify the logistic fungal growth model [47]:

(1) $$\ln[y/(K-y)]=\ln[y_{0}/(K-y_{0})]+r_{L}\cdot t$$

K (=Kmax) = maximum level of disease (y) or asymptote of disease progress curve, *y* = disease at time of observation, $y_{0}$ = level of disease at first observation, $r_{L}$ = rate of disease increase for the specific model and *t* = time interval being considered.

One-way analysis of variance (ANOVA) was also carried out to analyze the accumulation of fungal biomass using Duncan’s multiple range test through SPSS (SPSS 16.0, SPSS, Inc). Significant effects were set at *p* < 0.01. Data were checked for normality and the homogeneity of variances during the analysis.

## Supplementary Materials

The following are available online at https://www.mdpi.com/2076-0817/9/6/418/s1, Figure S1: *Bgt* development in resistant RIL CHR36 and susceptible RIL CHR32 at 4 and 10 dpi. Figure S2: *Bgt* penetration and haustoria formation in both resistant G305-3M and susceptible LDN, avoiding stomatal guard cell. Figure S3: The comparison of fungal biomass between the susceptible (CHR32 and LDN) and resistant (CHR36 and G305-3M) lines at the same time frame. Figure S4: Pretesting of H_2_O_2_ accumulation in young leaves of resistant G305-3M and susceptible LDN at 1 to 3 dpi inoculated with *Bgt*#70. Figure S5: Optimization of wheat germ agglutinin (WGA) chitin (WAC) fungal biomass measurement method and comparative amounts of fungal biomass accumulation within leaf tissues resistant RIL CHR36 and susceptible RIL CHR32. Table S1: Graphical genotypes of F_6_ recombinant inbred lines (RILs) derived from the cross of *T. dicoccoides* accession G305-3M with *T. durum* cultivar LDN. Table S2: A list of the publicly available markers, used in the current study, their locus name, type, primer sequence and fragment length in *Triticum durum* cv LDN and *Triticum dicoccoides* G305-3M. Table S3: Segregation ratio of the response to infection with isolate *Bgt#70* F_2_ in the progenies of the cross between the susceptible LDN and the resistant G305-3M, used for genetic mapping for *PmG3M*.
