# Supplementary material for: A Post-Haustorial Defense Mechanism is Mediated by the Powdery Mildew Resistance Gene, PmG3M, Derived from Wild Emmer Wheat
Source: Pathogens. 2020 May 28;9(6):418. doi: 10.3390/pathogens9060418 (PMC7350345; doi:10.3390/pathogens9060418)
Supplement: Supplementary file 1 [file pathogens-09-00418-s001.pdf]

Supplementary

Tables

Table S1

Table S1. Graphical genotypes of F_6 recombinant inbred lines (RILs) derived from the cross of *T. dicoccoides* accession G305-3M with *T. durum* cultivar LDN.

Markers / RILs and parents	*stm583*	Response to *Bgt#70*	*edm149*
CHR32	G	S	L
CHR36	L	R	R
G305-3M	G	R	G
LDN	L	S	L

Note: In this table, alleles highlighted in green were identical to those obtained for G305-3M, while alleles highlighted in yellow were identical to LDN. Resistance or susceptibility to *Bgt#70* is specified and highlighted, respectively, in green or yellow. The order of markers for delimiting the *PmG3M* region is based on the graphical genotyping of *PmG3M* according to Ben-David (2011).

Table S2

Table S2 A list of the publicly available markers, used in the current study, their locus name, type, primer sequence, and fragment length in *Triticum durum* cv LDN, and *Triticum dicoccoides* G305-3M.

Locus	Type	Primer sequence	Fragment size (bp)[a]		Reference
			LDN	G305-3M	
stm583	STM	5' cactgtgtagtactgctg 3'	305	313	Hayden et al 2006
edm149	EST-SSR	5' acacacacacacactctc 3'<br>5' atccacgccaagcagaag 3'<br>5' ctgtgggaagaagtgccttg 3'	209	207	Mullan et al. 2005

[a] Fragment size (bp) for the resistant *T. dicoccoides* accession G305-3M, which is the donor of *PmG3M*, and the susceptible *T. durum* cultivar Langdon (LDN).

Table S3

Table S3. Segregation ratio of the response to infection with isolate *Bgt#70* F_2 in the progenies of the cross between the susceptible LDN and the resistant G305-3M, used for genetic mapping for *PmG3M*.

Cross	F_2 resistant plants	F_2 susceptible plants	Expected ratio	Obtained ratio	$\chi 2$	*p* value*
LDN/ G305-3M	204	61	3:1	3.34:1	0.5547	0.4564

* *p* value for two tails test of significance by $\chi 2$ statistics with $df = 1$

Figures

Figure S1

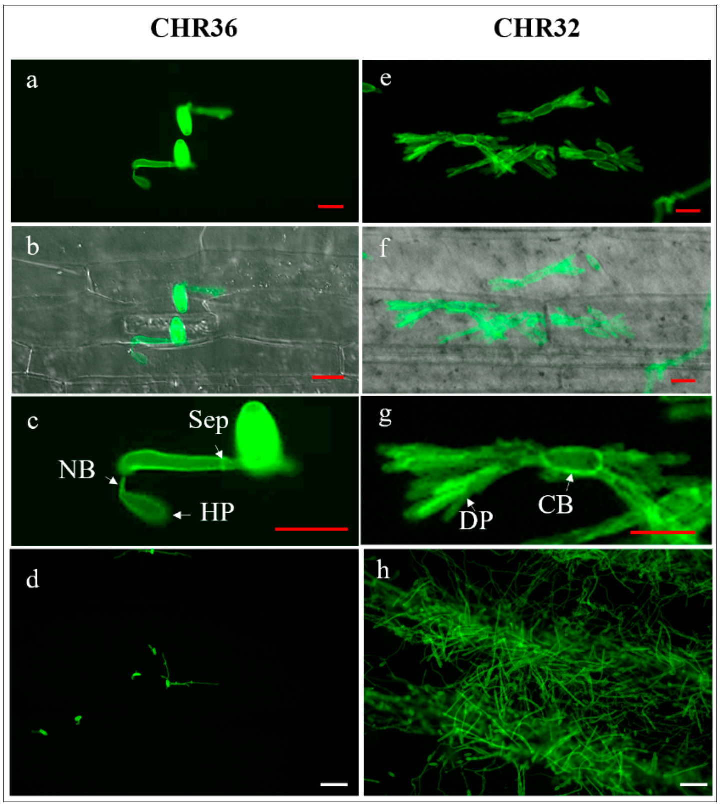

Figure S1. ***Bgt*** **development in resistant RIL CHR36 and susceptible RIL CHR32 at 4 and 10 dpi**. All samples were stained with wheat germ agglutinin (WGA) prior to observation under fluorescence microscopy. F_6 RILs were derived from G305-3M crossed with LDN by single seed descent (SSD) procedure. (**a**,**b**) germinating conidia, appressoria and haustorial primordia observed under fluorescence microscope (**a**) and superimposed on a bright field image (**b**) in CHR36 at 4 dpi; (**c**) high-resolution of the conidium with haustorial primordium at 4 dpi; (**d**) *Bgt* arrest of development in CHR36 at 10 dpi. (**e**,**f**) image of multiple mature haustoria observed in one epidermal cell under fluorescence microscope (**e**) and superimposed on a bright field image (**f**) in CHR32 at 4 dpi; (**g**) a matuer haustorium with central body and digitate processes in CHR32 at 4 dpi; (**h**) a large number of conidiophores produced in CHR32 at 10 dpi. CB: central body; DP: digitate processes (finger-like projections); HP: haustorial primordium; NB: neckband; Sep: septum. White scale bars are 100 μm, while red scale bars are 20 μm.

Figure S2

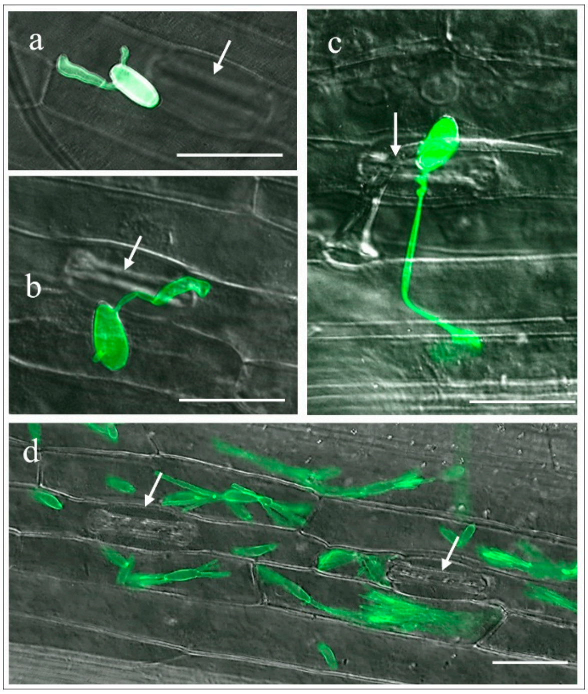

Figure S2. ***Bgt* penetration and haustoria formation in both resistant G305-3M and susceptible LDN, avoiding stomatal guard cell.** All samples were stained with wheat germ agglutinin (WGA) prior to observation under fluorescence microscopy. All micrographs were observed under superimposed of fluorescence and bright field. Panel (**a**) and (**b**) were G305-3M at 12 and 18 hpi respectively; panel (**c**) and (**d**) were LDN at 18 and 96 hpi respectively. Arrows indicate stomata. Scale bars are 50 μm.

Figure S3

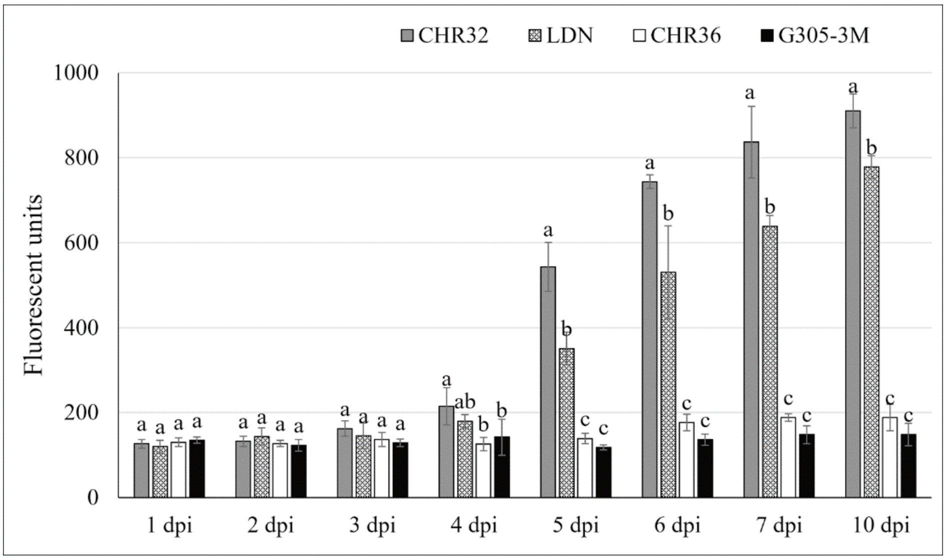

Figure S3. The comparison of fungal biomass between the susceptible (CHR32 and LDN) and resistant (CHR36 and G305-3M) lines at the same time frame. For each time point, different letters (a–c) indicate significant differences among the four lines based on Duncan's multiple range test ($p < 0.01$). Error bars denote standard deviation based on five biological replicates. dpi: days after inoculation.

Figure S4

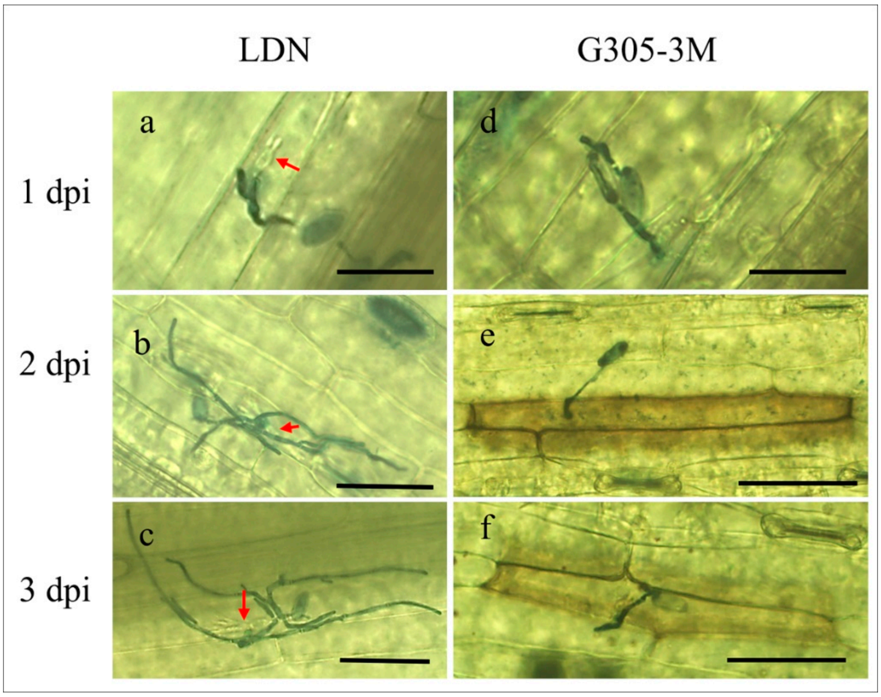

Figure S4. Pretesting of H_2O_2 accumulation in young leaves of resistant G305-3M and susceptible LDN at 1 to 3 dpi inoculated with *Bgt*#70. The leaf samples were stained with DAB-aniline blue and all micrographs were observed under a light microscope. Arrows indicate transparent haustoria without staining. Scale bars are 100 μm.

Figure S5

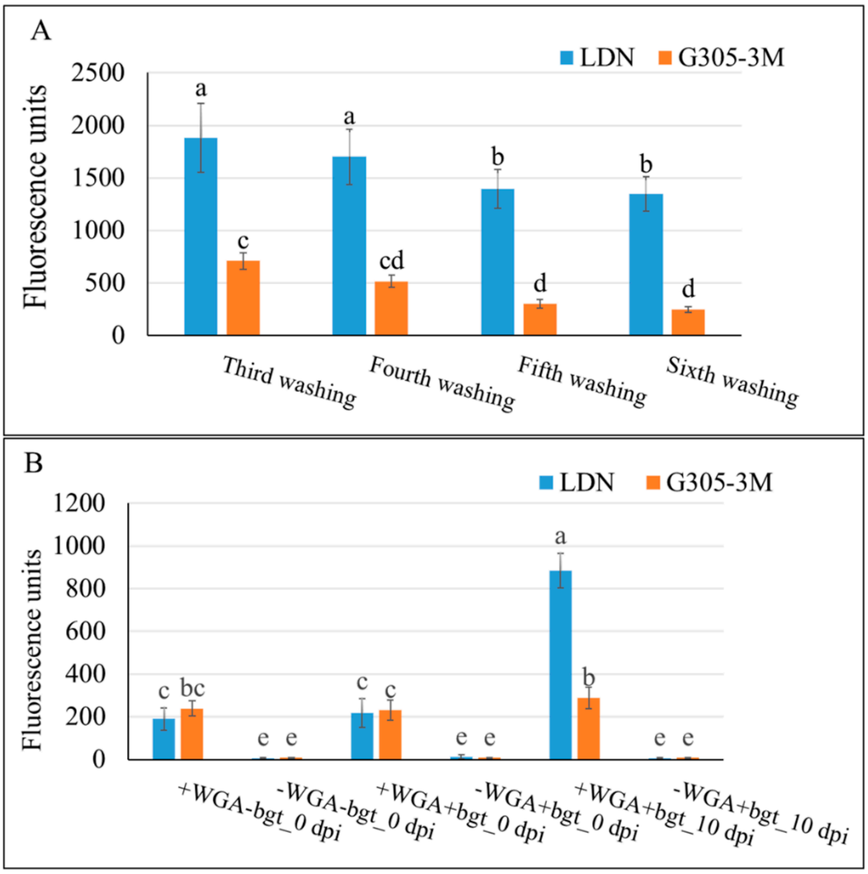

Figure S5. Optimization of wheat germ agglutinin (WGA) chitin (WAC) fungal biomass measurement method and comparative amounts of fungal biomass accumulation within leaf tissues resistant RIL CHR36 and susceptible RIL CHR32. Panel A: WAC measurements after different number of washing steps to remove extra WGA. Panel B: WAC measurements under different times post inoculation of *Bgt*. '+' and '–' indicate the measurement was performed with and without the followed issue, respectively. Error bars denote standard deviation based on five biological repeats. Different letters a–e indicate significant differences among the samples according to Duncan's multiple range test ($P < 0.05$).
